# Update on Trends in Placenta Accreta Syndrome and Its Impact on Maternal–Fetal Morbidity in the United States

**DOI:** 10.1177/26884844251378999

**Published:** 2025-09-24

**Authors:** Mulubrhan F. Mogos, Tara Maxa, John Watts, Unique J. Laylor, Sonja Hayden Emerson, Taneisha Gillyard Cheairs

**Affiliations:** ^1^ Center for Research Development and Scholarship, School of Nursing, Vanderbilt University, Nashville, Tennessee, USA.; ^2^ Texas A&M University College of Nursing, Bryan, Texas, USA.; ^3^ Department of Biomedical Sciences, Meharry Medical College, Nashville, Tennessee, USA.

**Keywords:** placenta accreta syndrome, pregnancy, cesarean section, pregnancy outcomes, prevalence, trends

## Abstract

**Background:**

Placenta accreta spectrum (PAS) is a life-threatening obstetric condition characterized by abnormal placental adherence to the uterine wall, leading to severe maternal morbidity and mortality. Rising cesarean delivery rates have contributed to its increasing prevalence, yet recent national data on PAS trends and disparities remain limited. This study provides updated estimates of PAS prevalence, racial and geographic disparities, and associated maternal–fetal outcomes using a large, nationally representative dataset from 2016 to 2022.

**Method:**

We conducted a retrospective cross-sectional analysis using the Nationwide Inpatient Sample (NIS), capturing 27,339,861 pregnancy-related hospitalizations. PAS cases (*n* = 36,310) were identified using International Classification of Disease, Tenth Revision, codes. Temporal trends were assessed using joinpoint regression. Survey logistic regression models estimated associations between PAS and maternal–fetal outcomes, adjusting for demographic, clinical, and hospital factors.

**Results:**

PAS prevalence increased significantly between 2016 and 2022 (annual percent change: 2.9%, *p* < 0.05), with notable increases among Black and White individuals. PAS was strongly associated with prior cesarean delivery, placenta previa, advanced maternal age, and comorbidities including hypertension, diabetes, and obesity. PAS significantly increased the risk of severe maternal morbidity, including hysterectomy (adjusted odds ratio [aOR] range: 52.2–151.3), blood transfusion (aOR range: 4.3–7.1), and preterm birth (aOR range: 2.4–3.2).

**Conclusions:**

These findings highlight the growing burden of PAS and the urgent need for prevention strategies, such as reducing unnecessary cesarean deliveries, promoting vaginal birth after cesarean when appropriate, enhancing prenatal screening, and ensuring multidisciplinary care. Addressing racial and geographic disparities in PAS diagnosis and outcomes is essential to improve maternal and neonatal health.

## Introduction

Placenta accreta spectrum (PAS), a pathological adherence of the placenta to the uterine wall, represents a critical obstetric complication with varying degrees of severity: accreta (attachment to the myometrium without decidua), increta (invasion into the myometrium), and percreta (invasion into surrounding organs).^1,2^ PAS is a significant contributor to maternal morbidity and mortality, accounting for up to 7% of maternal deaths globally.^1,3^ The condition is a leading cause of peripartum hysterectomy, often to prevent the risk of severe hemorrhage and complications such as disseminated intravascular coagulation and multiorgan failure.^2,4^

Despite its relative infrequency, PAS is an escalating health concern as cesarean delivery rates, one of the primary risk factors due to scar formation, continue to rise globally.^2,5^ In the United States, contemporary data have highlighted a worrying trend: the incidence of PAS has increased from historical rates of 1 in 2500 deliveries in the 1980s^6^ to 1 in 313 by the late 2010s, reflecting both rising cesarean deliveries and improved diagnostic capabilities.^4,5^ However, much of the U.S. data on PAS is outdated^4,5^ with a notable lack of comprehensive, nationally representative analyses exploring recent PAS trends, racial and geographic disparities in PAS, and associated outcomes. The most recent data on PAS for the United States limited the analysis to those with prior history of cesarean section.^5^

This study seeks to address these gaps by leveraging a nationally representative sample of inpatient hospital discharges in the United States to: (1) provide an updated assessment of the prevalence temporal trends and associated racial and geographical disparities of PAS from 2016 to 2022, and (2) analyze maternal–fetal outcomes associated with PAS. By providing contemporary insights into the epidemiology and clinical burden of PAS, this research aims to inform strategies for prevention, management, and health care planning to mitigate the rising burden of this deadly obstetric complication.

## Method

### Study design and data source

A retrospective cross-sectional analysis was conducted using the U.S. Nationwide Inpatient Sample (NIS) from 2016 to 2022, which is part of the Healthcare Cost and Utilization Project (HCUP). The NIS is the largest publicly available all-payer database of inpatient hospitalizations in the United States. Since the fourth quarter of 2015, the NIS has utilized International Classification of Disease, Tenth Revision, Clinical Modification (ICD-10-CM/PCS) codes for diagnoses and procedures. As the NIS data are de-identified and publicly accessible, this study was deemed exempt by the institutional review board.

### Study population

The study included pregnancy-related hospitalizations among women aged 13–49 years. Pregnancy-related hospitalizations were identified using the ICD-10-CM algorithm developed by Adman et al.^7^ All diagnosis and procedure fields were scanned for ICD-10 codes related to PAS diagnosis and relevant clinical and behavioral factors, including tobacco and alcohol use. The ICD-10 codes used in this study are detailed in Supplementary Table S1.

### Variables

Sociodemographic characteristics available in the NIS included age, race/ethnicity, primary payer, and median household income. Age was categorized into five groups: 13–24, 25–29, 30–34, 35–39, and 40–49 years. Race/ethnicity was grouped as Hispanic or non-Hispanic, with non-Hispanic individuals further classified as White, African American/Black, Asian/Pacific Islander, Native American, or other/unreported. Insurance status was designated as government (Medicare/Medicaid), private, and other (*e.g.*, self-pay, charity). Community-level socioeconomic status was approximated using ZIP-code-level median household income quartiles. Hospital characteristics, including geographic region (Northeast, Midwest, South, and West) and urban/rural status, were also analyzed.

### Statistical analysis

Descriptive statistics, including frequencies and percentages, were used to summarize patient and hospital characteristics by PAS diagnosis. PAS prevalence per 100,000 pregnancy-related hospitalizations was calculated and stratified by race/ethnicity, geographic region, and hospital urbanicity/teaching status. Differences in demographic, behavioral, and clinical characteristics between hospitalizations with and without PAS diagnoses were assessed.

Temporal trends in PAS prevalence stratified by maternal race, maternal age, and geographic location from 2016 to 2022 were analyzed using joinpoint regression. To provide more context to the trends in PAS, we also investigated trends of cesarean section and placenta previa, two common risk factors of PAS. This method initially models the annual prevalence as a straight line (zero joinpoints) and iteratively adds joinpoints to identify statistically significant changes in trends using Monte Carlo permutation tests. The final model reports each joinpoint and corresponding annual percent change.

Survey-weighted logistic regression was used to estimate adjusted odds ratios (aORs) for PAS and associated outcomes. Models adjusted for key demographic, behavioral, and clinical covariates, including maternal age, income quartile, tobacco use, alcohol use, cannabis use, opioid use, other substance use, and prior cesarean section history. We also included a composite variable for severe maternal morbidity (SMM), defined according to Center for Disease Control and Prevention (CDC) criteria, excluding blood transfusion (which was analyzed separately as a primary outcome). The SMM composite included eclampsia, acute renal failure, adult respiratory distress syndrome, disseminated intravascular coagulation, heart failure during procedure or surgery, puerperal cerebrovascular disorders, pulmonary edema, sepsis, shock, air or thrombotic embolism, cardiac arrest/ventricular fibrillation, conversion of cardiac rhythm, and hysterectomy.

All analyses were performed using SAS version 9.4 (SAS Institute, Inc., Cary, NC) and Joinpoint Regression Program version 4.8.0.1. Two-sided tests with a 5% significance level were used. Per HCUP data suppression rules, tables and figures exclude counts based on 10 or fewer events.^8^

## Results

**FIG. 1.**
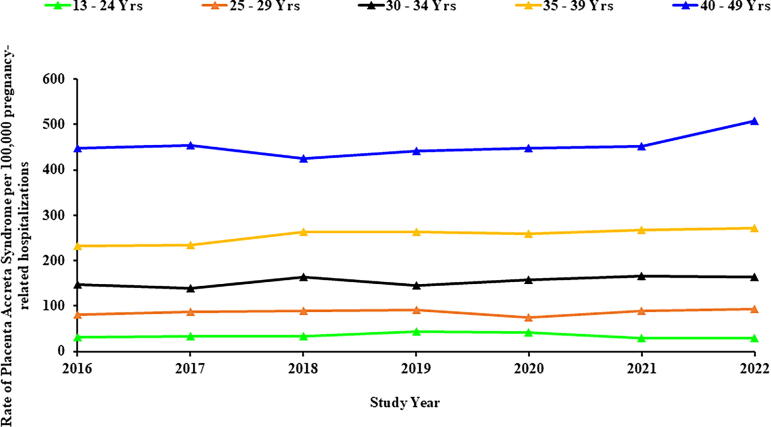
Trends in placenta accreta syndrome stratified by maternal age group (NIS: 2016–2022). X-axis: Study year. Y-axis: Rate of placenta accreta syndrome per 100,000 pregnancy-related hospitalizations. NIS, Nationwide Inpatient Sample.

Among the 27,339,861 pregnancy-related hospitalizations during the study period, 36,310 (0.133%) or 133 cases of PAS per 100,000 pregnancy-related hospitalizations were identified. Table 1 summarizes the association of demographic, behavioral, and hospital characteristics with PAS risk stratified by maternal race group. Maternal age was significantly associated with PAS risk across all racial groups. Compared to individuals aged 13–24 years, the odds of PAS increased with age, with the highest risk observed in the 40–49 age group (White: OR = 12.5, 95% confidence interval [CI]: 10.5–15.0; Black: OR = 18.8, 95% CI: 14.3–24.8; Hispanic: OR = 10.7, 95% CI: 8.4–13.7; Asian/Pacific Islanders: OR = 10.8, 95% CI: 6.0–19.3; Native American: OR = 7.0, 95% CI: 2.6–18.9; other/unreported: OR = 14.3, 95% CI: 9.8–20.9) (Table 1 and Fig. 1).

**Table 1.** Bivariate Odds Ratio and Associated 95% Confidence Interval Summarizing the Distribution of Placenta Accreta Syndrome by Demographic, Behavior, Payer, and Hospital Characteristics among Pregnancy-Related Hospitalizations Across Racial Groups: Nationwide Inpatient Sample, United States (2016–2022)

Characteristics Total *N* = 27,339,861	Total cases of PAS = 36,310					
	White *N* (%) cases	Black *N* (%) cases	Hispanic *N* (%) cases	Asian/Pacific Islanders *N* (%) cases	Native American *N* (%) cases	Other/unreported *N* (%) cases
	16,790 (46.2)	6,000 (16.5)	7,675 (21.1)	2,240 (6.2)	390 (1.1)	3,215 (8.9)
	OR (95% CI)	OR (95% CI)	OR (95% CI)	OR (95% CI)	OR (95% CI)	OR (95% CI)
Age group in years						
13–24	***Reference***	***Reference***	***Reference***	***Reference***	***Reference***	***Reference***
25–29	**2.3 (1.9–2.7)**	**3.8 (2.9–4.9)**	**2.7 (2.2–3.4)**	1.1 (0.6–2.1)	1.3 (0.6–2.9)	**2.2 (1.6–3.2)**
30–34	**4.0 (3.4–4.6)**	**7.0 (5.5–9.0)**	**5.5 (4.5–6.7)**	**2.1 (1.2–3.7)**	**3.0 (1.5–6.1)**	**4.5 (3.2–6.3)**
35–39	**6.8 (5.8–7.9)**	**11.6 (9.0–14.9)**	**7.9 (6.4–9.6)**	**4.5 (2.6–8.0)**	**5.8 (2.8–11.8)**	**7.2 (5.1–10.1)**
40–49	**12.5 (10.5–15.0)**	**18.8 (14.3–24.8)**	**10.7 (8.4–13.7)**	**10.8 (6.0–19.3)**	**7.0 (2.6–18.9)**	**14.3 (9.8–20.9)**
Alcohol use (yes)	1.5 (0.8–2.7)	0.6 (0.1–2.2)	1.0 (0.2–3.8)	0.0 (0.0–0.0)	1.1 (0.1–7.8)	0.9 (0.1–6.0)
Tobacco use (yes)	**1.5** **(1.4–1.7)**	**1.4** **(1.2–1.7)**	**2.0** **(1.4–2.7)**	**2.8** **(1.5–5.3)**	**1.7** **(0.9–2.9)**	**2.3** **(1.7–3.1)**
Cannabis use	0.8 (.06–1.1)	0.9 (0.7–1.3)	1.8 (0.7–1.9)	1.0 (0.1–6.9)	0.3 (0.0–1.8)	1.7 (1.0–2.9)
Substance use	1.1 (0.8–1.3)	1.0 (0.7–1.3)	**1.8** **(1.2–2.6)**	0.0 (0.0–0.0)	0.8 (0.3–2.1)	**1.8** **(1.1–3.0)**
Opioid use	**1.7** **(1.4–2.2)**	0.8 (0.3–1.9)	1.8 (1.0–3.4)	2.0 (0.3–14.0)	1.5 (0.5–4.9)	2.2 (1.0–4.9)
Household income						
0–25^th^ percentile	**0.8** **(0.7–0.9)**	**0.6** **(0.5–0.7)**	1.0 (0.8–1.2)	1.1 (0.8–1.5)	2.0 (0.8–5.0)	1.0 (0.8–1.3)
26^th^–50^th^ percentile	**0.8** **(0.7–0.9)**	**0.6** **(0.5–0.8)**	0.9 (0.8–1.1)	0.9 (0.7–1.3)	1.9 (0.7–5.1)	1.0 (0.8–1.2)
51^th^–75^th^ percentile	**0.8** **(0.7–0.9)**	**0.7** **(0.6–0.8)**	1.0 (0.8–1.1)	1.0 (0.9–1.3)	1.4 (0.5–3.8)	1.0 (0.8–1.2)
76^th^–100^th^ percentile	***Reference***	***Reference***	***Reference***	***Reference***	***Reference***	***Reference***
Hospital region						
Northeast	1.2 (1.0–1.3)	**1.3** **(1.1–1.6)**	**1.4** **(1.2–1.6)**	1.1 (0.8–1.5)	1.1 (0.4–3.3)	**1.3** **(1.1–1.7)**
Midwest	1.1 (1.0–1.2)	1.0 (0.9–1.2)	1.2 (0.9–1.4)	1.1 (0.8–1.6)	1.4 (0.7–2.8)	1.1 (0.9–1.4)
South	***Reference***	***Reference***	***Reference***	***Reference***	***Reference***	***Reference***
West	**1.2** **(1.1–1.3)**	1.3 (1.0–1.6)	1.2 (1.0–1.3)	1.2 (0.9–1.6)	1.4 (0.8–2.4)	**1.4** **(1.1–1.8)**
Hospital bedsize						
Small	**0.5** **(0.4–0.6)**	**0.5** **(0.4–0.6)**	**0.5** **(0.4–0.6)**	**0.4** **(0.3–0.6)**	0.6 (0.3–1.1)	**0.5** **(0.4–0.6)**
Medium	**0.6** **(0.6–0.7)**	**0.6** **(0.6–0.7)**	**0.6** **(0.6–0.7)**	**0.6** **(0.5–0.8)**	0.6 (0.3–1.0)	**0.6** **(0.5–0.7)**
Large	***Reference***	***Reference***	***Reference***	***Reference***	***Reference***	***Reference***
Hospital location/teach						
Rural	1.1 (0.9–1.3)	0.8 (0.5–1.3)	0.8 (0.5–1.1)	1.0 (0.4–2.4)	1.0 (0.4–2.7)	0.9 (0.6–1.4)
Urban nonteaching	***Reference***	***Reference***	***Reference***	***Reference***	***Reference***	***Reference***
Urban teaching	**2.1** **(1.9–2.4)**	**2.3** **(1.8–2.9)**	**1.8** **(1.5–2.1)**	**1.5** **(1.1–2.0)**	2.4 (1.0–5.4)	**1.7** **(1.3–2.3)**

Bold = statistically significant at *p* ≤ 0.05.

CI, confidence interval; OR, odds ratio.

Tobacco use was associated with increased odds of PAS across all racial groups, with the highest risk observed among Asian/Pacific Islanders (OR = 2.8, 95% CI: 1.5–5.3) and other/unreported (OR = 2.3, 95% CI: 1.7–3.1). Alcohol and cannabis use showed mixed associations with PAS, with no significant associations among certain racial groups. Compared to the reference group (highest quartile household income), individuals in the lowest household income brackets had reduced odds of PAS, particularly among Black (OR = 0.6, 95% CI: 0.5–0.7) and White (OR = 0.8, 95% CI: 0.7–0.9) populations. However, the association of PAS with household income was not statistically significant for Hispanic, Asian/Pacific Islanders, Native Americans, and other racial groups.

The odds of PAS varied by hospital region and setting. Compared to the South region of the United States, pregnancy-related hospitalizations in the Northeast and West regions were associated with increased odds of PAS. The highest risk was observed among Hispanic individuals in the Northeast (OR = 1.4, 95% CI: 1.2–1.6) and other/unreported individuals in the West (OR = 1.4, 95% CI: 1.1–1.8). In an adjusted analysis controlling for prior cesarean section, regional differences in PAS persisted: compared to the West (reference), the odds of PAS remained significantly lower in the Midwest (adjusted OR = 0.86, 95% CI: 0.79–0.94) and South (adjusted OR = 0.77, 95% CI: 0.71–0.84), while there was no significant difference for the Northeast (adjusted OR = 0.99, 95% CI: 0.90–1.10). Compared to hospitals with large number of beds, hospitals with smaller number of beds are less likely to treat patients with PAS across all racial groups, except for Native Americans, where the odds were not statistically significant. As expected, patients with PAS are more likely to be treated in urban teaching hospitals compared to rural or urban nonteaching hospitals, likely reflecting referral patterns for complex obstetric care to centers with specialized expertise and multidisciplinary resources.

Table 2 summarizes the association of selected clinical morbidities with PAS risk stratified by maternal race group. We found statistically significant association between PAS and diagnosis of anxiety across all racial groups, with the highest odds among Asian/Pacific Islanders (OR: 2.5, 95% CI: 1.6–3.8) and Native Americans (OR: 2.5, 95% CI: 1.4–4.6). Patients with PAS were also more likely to have diagnosis of depression among White (OR: 1.5, 95% CI: 1.3–1.7), Hispanic (OR: 1.6, 95% CI: 1.2–2.0), and Asian/Pacific Islander pregnant individuals (OR: 2.5, 95% CI: 1.5–4.1). No significant association was found between bipolar disorder and PAS in any racial group. Chronic obstructive pulmonary disease (COPD) and asthma were significantly associated with PAS among multiple racial groups. The highest odds were observed among Native American individuals for COPD (OR: 2.2, 95% CI: 1.2–4.1) and asthma (OR: 2.2, 95% CI: 1.2–4.2).

**Table 2.** Bivariate Odds Ratio and Associated 95% Confidence Interval Summarizing the Distribution of Placenta Accreta Syndrome by Clinical Morbidities Among Pregnancy-Related Hospitalizations across Racial Groups: Nationwide Inpatient Sample, United States (2016–2022)

Characteristics Total *N* = 27,339,861	Total cases of PAS = 36,310					
	White *N* (%) cases	Black *N* (%) cases	Hispanic *N* (%) cases	Asian/Pacific Islanders *N* (%) cases	Native American *N* (%) cases	Other/unreported *N* (%) cases
	16,790 (46.2)	6,000 (16.5)	7,675 (21.1)	2,240 (6.2)	390 (1.1)	3,215 (8.9)
	OR (95% CI)	OR (95% CI)	OR (95% CI)	OR (95% CI)	OR (95% CI)	OR (95% CI)
Bipolar disorder	1.4 (1.0–1.8)	1.2 (0.8–1.9)	1.6 (0.9–2.8)	0.0 (0.0–0.0)	0.0 (0.0–0.0)	1.6 (0.8–3.2)
Anxiety	**1.4** **(1.3–1.6)**	1.1 (0.9–1.5)	**1.5** **(1.2–1.9)**	**2.5** **(1.6–3.8)**	**2.5** **(1.4–4.6)**	**1.7** **(1.3–2.3)**
Depression	**1.5** **(1.3–1.7)**	1.0 (0.8–1.4)	**1.6** **(1.2–2.0)**	**2.5** **(1.5–4.1)**	2.1 (.0–4.1)	1.3 (0.9–1.9)
COPD	**1.5** **(1.3–1.7)**	1.2 (1.0–1.4)	**1.3** **(1.1–1.6)**	1.3 (0.8–2.1)	**2.2** **(1.2–4.1)**	**1.5** **(1.1–2.1)**
Asthma	**1.5** **(1.3–1.7)**	1.2 (1.0–1.4)	**1.3** **(1.1–1.7)**	1.3 (0.8–2.0)	**2.2** **(1.2–4.2)**	**1.5** **(1.1–2.1)**
Obstructive sleep apnea	**3.1** **(1.9–5.0)**	**2.7** **(1.5–4.8)**	**7.0** **(3.8–12.8)**	**1.9** **(0.3–13.3)**	0.0 (0.0–0.0)	**9.8** **(4.7–20.7)**
Diabetes mellites	1.3 (0.9–1.7)	1.3 (0.9–1.9)	**2.2** **(1.7–3.0)**	**3.4** **(2.1–5.6)**	**3.0** **(1.2–7.8)**	**2.0** **(1.2–3.4)**
Hypertension	**1.5** **(1.3–1.7)**	**1.8** **(1.5–2.1)**	**1.9** **(1.5–2.3)**	1.6 (1.0–2.5)	1.1 (0.4–2.9)	**1.5** **(1.1–2.2)**
Coronary artery disease	**4.2** **(2.5–7.8)**	1.8 (0.7–4.8)	1.0 (0.1–7.1)	**8.9** **(2.1–36.2)**	0.0 (0.0–0.0)	**8.5** **(3.2–22.9)**
Obesity	**1.3** **(1.2–1.4)**	**1.6** **(1.4–1.9)**	**1.5** **(1.3–1.7)**	1.1 (0.7–1.6)	1.8 (1.0–3.2)	**1.8** **(1.4–2.4)**
Died	**16.5** **(7.3–37.0)**	0.0 (0.0–0.0)	**10.7** **(2.7–42.9)**	**30.4** **(7.4–124.7)**	0.0 (0.0–0.0)	0.0 (0.0–0.0)
Severe maternal morbidity	**63.3** **(58.9–67.9)**	**46.1** **(41.0–51.9)**	**76.9** **(69.2–85.5)**	**65.5** **(54.1–79.5)**	**34.1** **(22.0–52.8)**	**63.1** **(54.2–73.9)**

Bold = statistically significant at *p* ≤ 0.05.

COPD, chronic obstructive pulmonary disease.

Obstructive sleep apnea showed a strong association with PAS, with the highest odds among Hispanic individuals (OR: 7.0, 95% CI: 3.8–12.8) and other/unreported individuals (OR: 9.8, 95% CI: 4.7–20.7). In sensitivity analyses adjusting for both obesity and prior cesarean section history, obstructive sleep apnea (OSA) remained independently associated with increased odds of PAS. Overall, individuals with OSA had nearly threefold higher adjusted odds of PAS compared to those without OSA (aOR: 2.94; 95% CI: 2.20–3.93). This association was consistent across racial groups, remaining significant among Black individuals (aOR: 2.49; 95% CI: 1.52–4.08), White individuals (aOR: 2.00; 95% CI: 1.09–3.64), and Asian/Pacific Islander individuals (aOR: 4.93; 95% CI: 2.65–9.19). Among Hispanic individuals, the association did not reach statistical significance (aOR: 1.72; 95% CI, 0.25–11.91). For individuals classified as other/unreported race, the association was strongest (aOR: 7.20; 95% CI: 3.35–15.44). These results suggest that the observed association between OSA and PAS is not fully explained by confounding due to obesity or prior cesarean deliveries. Diabetes mellitus was significantly associated with PAS among Hispanic (OR: 2.2, 95% CI: 1.7–3.0), Asian/Pacific Islanders (OR: 3.4, 95% CI: 2.1–5.6), and Native Americans (OR: 3.0, 95% CI: 1.2–7.8).

Pregnant individuals with PAS were more likely to have diagnosis of hypertension across all racial groups, with the highest odds among Black (OR: 1.8, 95% CI: 1.5–2.1) and Hispanic individuals (OR: 1.9, 95% CI: 1.5–2.3). Coronary artery disease showed an especially strong association with PAS among Asian/Pacific Islanders (OR: 8.9, 95% CI: 2.1–36.2) and other/unreported individuals (OR: 8.5, 95% CI: 3.2–22.9). Obesity was significantly associated with PAS across all racial groups, with the highest odds among Native American (OR: 1.8, 95% CI: 1.0–3.2) and other/unreported individuals (OR: 1.8, 95% CI: 1.4–2.4).

**FIG. 2.**
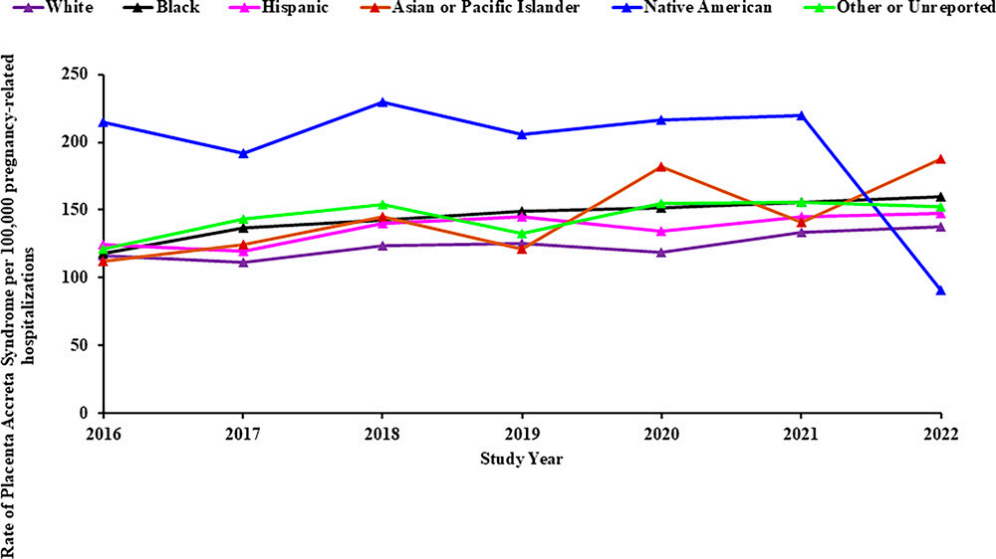
Trends in placenta accreta syndrome stratified by maternal race (NIS: 2016–2022). X-axis: Study year. Y-axis: Rate of placenta accreta syndrome per 100,000 pregnancy-related hospitalizations.

PAS rates varied across racial and ethnic groups, with a statistically significant increase among Black (Annual Percent Change [APC]: 4.4% [95% CI: 2.0, 7.0]) and White (APC: 3.0% [95% CI: 1.1, 4.7]) pregnancy-related hospitalizations. Hispanic, Asian/Pacific Islanders, and other/unreported racial groups also experienced an increase, though it did not reach statistical significance. No clear trend was observed for Native American (despite the sharp decline in PAS rate from 2021 to 2022) or other/unreported groups (Fig. 2).

**FIG. 3.**
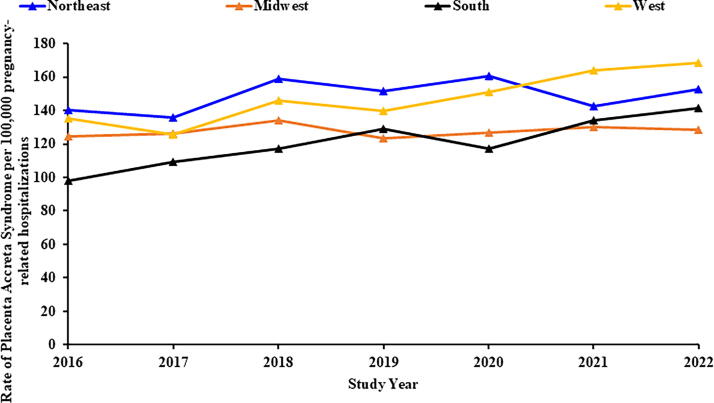
Trends in placenta accreta syndrome stratified by geographic region (NIS: 2016–2022). X-axis: Study year. Y-axis: Rate of placenta accreta syndrome per 100,000 pregnancy-related hospitalizations.

PAS rates increased most significantly in the South (APC: 5.5% [95% CI: 3.1, 8.0]), while no significant change was observed in the West, Northeast, and Midwest regions. PAS rates increased significantly among pregnant individuals in the 35–39 age category (APC: 2.5% [95% CI: 1.2, 3.9]). No significant change was observed among younger age groups or those in the 40–49 years age group category (Fig. 3).

**FIG. 4.**
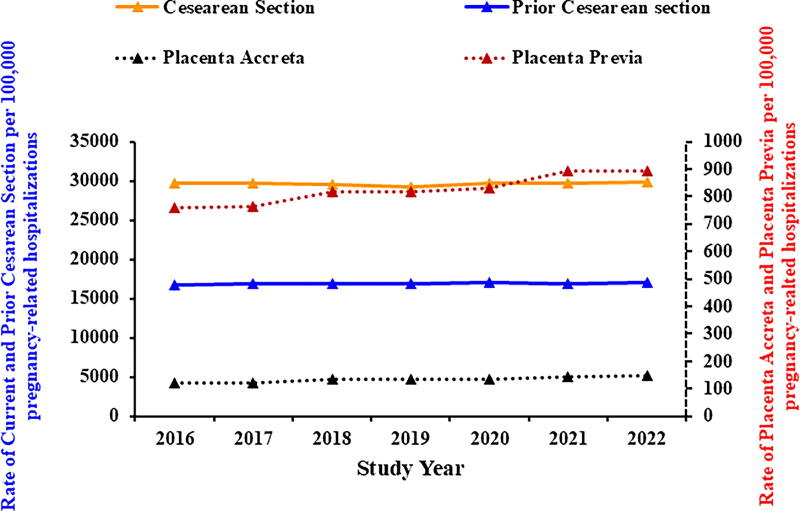
Trends in placenta accreta and placenta previa (broken line), as well as prior cesarean section and current cesarean section (full line): NIS: 2016–2022. X-axis: Study year. Y-axis (left side of the figure, full line): Rate of current and prior cesarean section per 100,000 pregnancy-related hospitalizations. Y-axis (right side of the figure, broken line): Rate of placenta accreta and placenta previa per 100,000 pregnancy-related hospitalizations.

Between 2016 and 2022, the rate of PAS showed a statistically significant increase (APC= 2.9% [95% CI: 1.8, 4.0]), whereas placenta previa, a condition known to co-occur with PAS often, exhibited minimal change with no statistically significant trend (APC = 0.2 [95% CI: −0.1, 0.4]). Concurrently, prior cesarean section rates increased during the study period, with a statistically significant APC of 3.4% (95% CI: 2.2, 4.6), reflecting a growing number of pregnancies complicated by prior cesarean section, a major risk factor for PAS (Fig. 4).

Women diagnosed with PAS had significantly higher odds of adverse maternal outcomes across racial groups. Hysterectomy was significantly more likely among White (aOR: 137.1, 95% CI: 105.7–177.7), Black (aOR: 151.3, 95% CI: 106.3–215.3), Hispanic (aOR: 317.0, 95% CI: 258.3–389.1), and Asian/Pacific Islander individuals (aOR: 431.1, 95% CI: 262.6–707.7). PAS was significantly associated with uterine repair across all racial groups, with the highest odds among Native American individuals (aOR: 42.4, 95% CI: 15.2–118.5) and Black individuals (aOR: 30.9, 95% CI: 22.7–42.1) (Table 3).

**Table 3.** Adjusted Odds Ratios, and 95% Confidence Intervals for the Association Between Placenta Accreta Syndrome and Maternal and Fetal Outcomes: Nationwide Inpatient Sample, United States (2016–2022)

Outcome	Total cases of PAS = 36,310											
	White *N* (%) case		Black *N* (%) cases		Hispanic *N* (%) cases		Asian/pacific islanders *N* (%) cases		Native American *N* (%) cases		Other/unreported *N* (%) cases	
	16,790 (46.2)		6,000 (16.5)		7,675 (21.1)		2,240 (6.2)		390 (1.1)		3,215 (8.9)	
	Crude OR (95% CI)	^a^aOR (95% CI)	Crude OR (95% CI)	^a^aoR (95% CI)	Crude OR (95% CI)	^a^aOR (95% CI)	Crude OR (95% CI)	^a^aOR (95% CI)	Crude OR (95% CI)	^a^aOR (95% CI)	Crude OR (95% CI)	^a^aOR (95% CI)
Hysterectomy	**540.1** **(499–584.5)**	**137.1** **(105.7–177.7)**	**322.7** **(284.7–365.9)**	^***^	**595.1** **(530.3–667.8)**	**151.3** **(106.3–215.3)**	**317** **(258.3–389.1)**	**52.2** **(30.94–88)**	**431.1** **(262.6–707.7)**	>^***^	**474.3** **(398.5–564.5)**	**127.3** **(72.2–224.6)**
Uterine repair	**126.9** **(109.3–147.2)**	**26.4** **(22.1–31.6)**	**116.1** **(88.9–151.6)**	**30.9** **(22.7–42.1)**	**145.7** **(117.4–180.9)**	**23.6** **(18.1–30.7)**	**104.1** **(69.9–154.9)**	**22.7** **(14.3–35.9)**	**129.5** **(46.9–357.3)**	**42.4** **(15.2–118.5)**	**108.2** **(75.2–155.6)**	**18.6** **(12.0–28.7)**
Breech presentation	**3.2** **(2.9–3.6)**	**2.6** **(2.4–2.9)**	**4.8** **(4.0–5.6)**	**3.3** **(2.8–3.9)**	**5.8** **(5.1–6.7)**	**4.2** **(3.7–4.9)**	**4.3** **(3.3–5.5)**	**3.2** **(2.4–4.1)**	**3.5** **(1.8–6.9)**	**2.6** **(1.3–5.3)**	**4.6** **(3.7–5.8)**	**3.4** **(2.7–4.3)**
PPROM	**2.2** **(1.9–2.5)**	**2.1** **(1.8–2.5)**	**2.1** **(1.7–2.6)**	**2.2** **(1.8–2.8)**	**2.5** **(2.0–3.1)**	**2.4** **(1.9–3.0)**	1.5 (0.9–2.4)	1.2 (0.8–2.0)	2.1 (0.8–5.0)	2.0 (0.8–4.9)	**2.1** **(1.5–2.9)**	**2.0** **(1.4–2.8)**
Fetal growth restriction	**1.6** **(1.4–1.9)**	**1.8** **(1.6–2.1)**	1.3 (1.0–1.6)	**1.6** **(1.3–1.9)**	1.1 (0.8–1.5)	1.2 (0.9–1.6)	1.4 (1.0–2.1)	**1.5** **(1.1–2.2)**	1.1 (0.4–3.6)	1.1 (0.4–3.5)	1.2 (0.9–1.8)	1.3 (0.9–1.9)
≥37 weeks of gestation	**0.1** **(0.1–0.1)**	**0.3** **(0.3–0.3)**	**0.2** **(0.1–0.2)**	**0.4** **(0.4–0.5)**	**0.1** **(0.1–0.1)**	**0.2** **(0.2–0.3)**	**0.1** **(0.1–0.1)**	**0.2** **(0.2–0.3)**	**0.2** **(0.1–0.3)**	**0.4** **(0.3–0.8)**	**0.1** **(0.1–0.1)**	**0.3** **(0.2–0.3)**
34–36 weeks of gestation	**5.4** **(5.1–5.9)**	**4.5** **(4.2–4.9)**	**5.0** **(4.4–5.6)**	**4.7** **(4.2–5.4)**	**5.5** **(5.0–6.2)**	**4.4** **(4.0–4.9)**	**5.9** **(4.8–7.3)**	**4.5** **(3.7–5.5)**	**2.8** **(1.6–4.8)**	**2.3** **(1.3–4.0)**	**5.8** **(4.9–6.9)**	**4.6** **(3.9–5.35)**
32–33 weeks of gestation	**6.1** **(5.4–6.9)**	**3.6** **(3.2–4.1)**	**4.7** **(3.9–5.6)**	**3.2** **(2.7–3.9)**	**7.7** **(6.6–9.0)**	**4.0** **(3.4–4.8)**	**8.7** **(6.4–11.8)**	**4.8** **(3.4–6.6)**	**8.8** **(4.9–15.8)**	**5.7** **(3.1–10.6)**	**7.8** **(6.1–10.0)**	**4.3** **(3.3–5.6)**
<32 weeks of gestion	**5.1** **(4.6–5.9)**	**2.1** **(1.9–2.4)**	**3.0** **(2.6–3.4)**	**1.6** **(1.4–1.9)**	**6.0** **(5.3–6.8)**	**2.1** **(1.8–2.4)**	**6.8** **(5.4–8.5)**	**2.5** **(1.9–3.2)**	**3.4** **(1.9–6.0)**	1.5 (0.8–2.8)	**4.7** **(3.8–5.8)**	**1.8** **(1.4–2.3)**
Blood transfusion	**27.8** **(25.4–30.3)**	**6.4** **(5.7–7.1)**	**20.9** **(18.4–23.9)**	**6.3** **(5.4–7.4)**	**28.7** **(25.7–32.1)**	**7.1** **(6.2–8.2)**	**28.9** **(23.5–35.7)**	**5.5** **(4.1–7.5)**	**10.8** **(6.0–19.6)**	**4.3** **(2.1–8.7)**	**25.2** **(21.1–30.1)**	**6.0** **(4.8–7.7)**
Intrauterine fetal demise	**1.7** **(1.2–2.6)**	0.7 (0.5–1.0)	**2.5** **(1.7–3.6**)	1.0 (0.7–1.5)	**1.9** **(1.1–3.2)**	0.7 (0.4–1.3)	1.9 (0.6–5.7)	0.9 (0.3–2.6)	2.0 (0.3–14.2)	0.8 (0.1–5.5)	1.1 (0.4–3.0)	0.4 (0.2–1.2)
Intrauterine growth restriction	**1.6** **(1.4–1.9)**	**1.8** **(1.6–2.1)**	1.3 (1.0–1.6)	**1.6** **(1.3–1.9)**	1.1 (0.8–1.5)	1.2 (0.9–1.6)	1.4 (1.0–2.1)	**1.5** **(1.1–2.2)**	1.1 (0.4–3.6)	1.1 (0.4–3.5)	1.2 (0.9–1.8)	1.3 (0.9–1.9)
Preterm	**2.9** **(2.6–3.2)**	**2.7** **(2.4–3.0)**	**2.5** **(2.1–2.9)**	**2.5** **(2.2–3.0)**	**3.8** **(3.3–4.4)**	**3.2** **(2.8–3.7)**	**3.0** **(2.3–4.1)**	**2.4** **(1.8–3.2)**	1.7 (0.8–3.7)	1.6 (0.7–3.7)	**3.2** **(2.6–4.1)**	**2.9** **(2.3–3.6)**
Prolonged hospital stay	**11.4** **(10.5–12.3)**	**2.7** **(2.4–3.0)**	**8.6** **(7.6–9.7)**	**2.2** **(1.9–2.6)**	**14.3** **(12.8–16.0)**	**2.8** **(2.4–3.2)**	**13.3** **(10.8–16.3)**	**2.7** **(2.1–3.6)**	**8.4** **(5.0–14.1)**	**2.4** **(1.3–4.7)**	**12.7** **(10.7–15.2)**	**2.8** **(2.2–3.5)**
Placenta previa	**66.4** **(61.6–71.7)**	**38.8** **(35.7–42.3)**	**88.2** **(78.0–99.7)**	**55.4** **(48.2–63.7)**	**113.8** **(102.5–126.2)**	**63.8** **(56.5–72.1)**	**72.1** **(59.4–87.5)**	**44.1** **(35.4–55.0)**	**77.0** **(48.1–123.2)**	**45.7** **(25.4–81.9)**	**80.0** **(68.0–94.1)**	**45.7** **(37.9–55.0)**
Prior cesarean section	**5.9** **(5.7–6.3)**	**5.5** **(5.1–5.9)**	**7.7** **(6.8–8.7)**	**7.2** **(6.3–8.1)**	**9.1** **(8.2–10.2)**	**8.5** **(7.6–9.6)**	**5.2** **(4.3–6.3)**	**4.5** **(3.7–5.5)**	**8.0** **(5.1–12.5)**	**8.0** **(5.0–12.9)**	**6.8** **(5.7–8.0)**	**6.1** **(5.1–7.3)**

Bold = statistically significant at *p* ≤ 0.05. *** = Data did not fit model.

^a^ Models adjusted for key demographic, behavioral, and clinical covariates, including maternal age, income quartile, tobacco use, alcohol use, cannabis use, opioid use, other substance use, and prior cesarean section history. We also included a composite variable for severe maternal morbidity (SMM), defined according to CDC criteria, excluding blood transfusion (which was analyzed separately as a primary outcome). The SMM composite included eclampsia, acute renal failure, adult respiratory distress syndrome, disseminated intravascular coagulation, heart failure during procedure or surgery, puerperal cerebrovascular disorders, pulmonary edema, sepsis, shock, air or thrombotic embolism, cardiac arrest/ventricular fibrillation, conversion of cardiac rhythm, and hysterectomy.

aOR, adjusted odds ratio; *N*, number; PAS, placenta accreta syndrome; PPROM, preterm premature rupture of membranes; PROM, premature rupture of membranes.

The likelihood of requiring a blood transfusion was significantly elevated for women diagnosed with PAS from all racial groups, with the highest odds observed among Hispanic (aOR: 7.1, 95% CI: 6.2–8.2), White (aOR: 6.4, 95% CI: 5.7–7.1), and Black individuals (aOR: 6.3, 95% CI: 5.4–7.4). Prolonged hospital stay was significantly associated with PAS across all racial groups, with the highest odds observed among Hispanic (aOR: 2.8, 95% CI: 2.4–3.2), other/unreported (aOR: 2.8, 95% CI: 2.2–3.5), and White individuals (aOR: 2.7, 95% CI: 2.4–3.0) (Table 3).

PAS was associated with an increased risk of adverse fetal outcomes, including abnormal presentations, growth restriction, and preterm birth. Breech presentation was significantly more common across all racial groups, with the highest odds among Hispanic individuals (aOR: 4.2, 95% CI: 3.7–4.9). Fetal growth restriction was significantly associated with PAS among White (aOR: 1.8, 95% CI: 1.6–2.1), Black (aOR: 1.6, 95% CI: 1.3–1.9), and Asian/Pacific Islander individuals (aOR: 1.5, 95% CI: 1.1–2.2). Preterm birth was significantly more common across all racial groups, with the highest odds among Hispanic (aOR: 3.2, 95% CI: 2.8–3.7), White (aOR: 2.7, 95% CI: 2.4–3.0), and Black individuals (aOR: 2.5, 95% CI: 2.2–3.0). Infants born to mothers with PAS were more likely to be delivered at 32–33 weeks of gestation, with Asian/Pacific Islanders having the highest adjusted odds (aOR: 4.8, 95% CI: 3.4–6.6) (Table 3).

Placenta accreta syndrome was strongly associated with delivery before 32 weeks of gestation, with Hispanic (aOR: 2.1, 95% CI: 1.8–2.4) and Asian/Pacific Islander individuals (aOR: 2.5, 95% CI: 1.9–3.2) having the highest odds. PAS was highly associated with placenta previa across all racial groups, with the highest adjusted odds observed in Hispanic (aOR: 63.8, 95% CI: 56.5–72.1) and Black individuals (aOR: 55.4, 95% CI: 48.2–63.7). Prior cesarean section was a significant risk factor for PAS, with the highest odds among Hispanic (aOR: 8.5, 95% CI: 7.6–9.6), Black (aOR: 7.2, 95% CI: 6.3–8.1), and Native American individuals (aOR: 8.0, 95% CI: 5.0–12.9) (Table 3).

## Discussion

The main findings of the study are: (1) the prevalence of PAS has increased over time, particularly among Black and White pregnant individuals; (2) PAS is strongly associated with increasing maternal age, with the highest risk observed among those aged 40–49 years across all racial groups; (3) SMM, maternal age, and prior cesarean section are among the most significant risk factors for PAS; and (4) PAS is associated with increased maternal morbidity, including blood transfusions, hysterectomy, and prolonged hospital stays, as well as adverse neonatal outcomes such as preterm birth and fetal growth restriction after adjusting for potential confounders.

Our findings on the increasing prevalence of PAS are consistent with prior epidemiological studies in the United States and globally. A previous analysis of national trends reported a steady rise in PAS cases, which has been attributed to the growing number of women undergoing multiple cesarean deliveries.^4,9–12^ This aligns with our finding that prior cesarean section remains one of the major risk factors for PAS, with significantly increased odds across all racial groups. Similar PAS trends have been observed in high-income countries,^13,14^ as well as low income countries^15^ where increasing rates of PAS parallel rising cesarean delivery rates. However, in some European countries with lower cesarean section rates, such as the Netherlands^16^ and other Nordic countries,^17^ PAS prevalence has remained relatively stable.

The strong association between maternal age and PAS risk in our study is well-documented in prior research. Globally, the birth rate among older women has increased exponentially in the 35–39 and 40–44 age groups.^1,18^ Older maternal age is linked to an increased likelihood of placental implantation abnormalities, which may be due to uterine scarring from previous deliveries^19^ and age-related changes in endometrial receptivity.^4,20^ Our results indicate that the risk of PAS is highest among individuals aged 40–49 years across all racial groups, a pattern consistent with prior U.S. hospital-based studies.^18,21^ This age-related risk underscores the need for targeted screening and management strategies, particularly among older pregnant individuals with additional risk factors.

We also identified racial and regional disparities in PAS prevalence. The highest increase was observed among Black and White pregnant individuals. This finding is consistent with previous studies that have reported racial disparities in PAS, possibly due to differences in healthcare access, prenatal care utilization, and underlying maternal health conditions.^22,23^ Regional variations in PAS prevalence, with higher odds observed in the Northeast and West, suggest potential differences in hospital practices, access to specialized care, or diagnostic approaches. A prior study has similarly reported global geographic disparities in PAS rates, particularly in regions with higher cesarean section prevalence.^24^ In sensitivity analyses adjusting for prior cesarean section, regional differences in PAS prevalence were attenuated but persisted, suggesting that variation in cesarean rates accounts for some but not all of the observed geographic disparities.

The association between PAS and obesity^25^ is supported by existing literature. However, the association of PAS with diabetes or hypertensive disorders of pregnancy has not been established, although there is a report from one study that shows inverse relationship between PAS and hypertensive disorders of pregnancy.^26^ Prior cesarean section and advanced maternal age are known risk factors of PAS. In our sensitivity analysis adjusting for obesity and prior cesarean section history, obstructive sleep apnea (OSA) remained significantly associated with higher odds of PAS, suggesting that the observed relationship is not fully explained by confounding from these known risk factors. This finding supports the hypothesis that OSA may represent an independent risk factor for PAS and warrants further investigation into potential underlying mechanisms. The strong association between OSA and PAS is a novel finding that warrants further investigation, as sleep apnea is an emerging risk factor for adverse pregnancy outcomes.^27^

Our findings confirm the high burden of morbidity associated with PAS, with notable racial and ethnic disparities in adverse outcomes, even after adjustment for potential confounders. The association between PAS and the high risk of hysterectomy observed in our study aligns with existing literature, which underscores hysterectomy as a common and often necessary intervention in PAS cases due to the invasive nature of the placental attachment.^3,13,28^ The significantly elevated adjusted odds ratio for hysterectomy across all racial groups highlights the severity of PAS regardless of demographic background. However, Hispanic women exhibited slightly higher adjusted odds ratios for hysterectomy, which may reflect variations in surgical decision-making processes or variations in rates of other morbid conditions.^29^

Our findings also indicate that PAS is associated with a significantly increased likelihood of the need for blood transfusion, uterine repair, and prolonged hospital stay, which are consistent with previous studies reporting the increased surgical complexity and risk of hemorrhage associated with PAS.^30,31^

In line with prior studies, we observed a strong association between PAS and adverse fetal outcomes, particularly preterm birth.^32,33^ The significantly increased odds of delivery before 37 weeks and a high proportion of deliveries occurring before 34 weeks highlight the critical need for improved prenatal surveillance and early intervention strategies for PAS-affected pregnancies.^21^ We also found an association between PAS and fetal growth restriction in White, Black, and Asian/Pacific Islander pregnant individuals. However, it is important to note that others did not find association between PAS and fetal growth restriction,^34,35^ and in our study, the association was not significant for pregnant individuals identified as Hispanic or of Other/Unreported race. Placenta previa, a well-established risk factor for PAS, was notably prevalent in our cohort, with Native American and Hispanic women showing the highest adjusted odds ratios. This finding is consistent with previous research highlighting the strong association between placenta previa and PAS.^35,36^

This study has several limitations. First, our data were derived from hospital-based records, which may be subject to coding errors and misclassification of PAS diagnoses. This limitation has been previously noted in studies using administrative databases.^4^ In addition, the broader denominator in our analysis (all pregnancy-related hospitalizations rather than delivery hospitalizations) likely contributes to the lower observed prevalence compared to prior data.^4^ Second, we could not account for detailed clinical variables, such as ultrasound findings or surgical reports, which are crucial for confirming PAS diagnosis. Third, while we identified significant racial and socioeconomic disparities, our data do not capture factors such as healthcare access, provider practices, and patient preferences that may influence PAS incidence. Finally, our study is limited by the inability to assess long-term maternal and neonatal outcomes beyond hospitalization.

Our findings highlight the increasing burden of PAS in the United States, with significant racial, regional, and socioeconomic disparities. The continued rise in PAS prevalence, particularly among older pregnant individuals and those with prior cesarean sections, underscores the urgent need for enhanced clinical screening and risk stratification strategies. From a clinical perspective, healthcare providers should prioritize early identification of PAS risk factors through routine obstetric screenings, particularly in high-risk populations. Increased utilization of magnetic resonance imaging and targeted ultrasonography in high-risk pregnancies may facilitate early diagnosis and improve perinatal outcomes.^37,38^ The implementation of standardized protocols for PAS management, including multidisciplinary care teams and hospital readiness plans, could improve maternal and neonatal outcomes.^5^ From a research standpoint, further studies are needed to investigate the biological mechanisms underlying PAS, particularly the role of comorbid conditions such as sleep apnea and cardiovascular disease. Prospective cohort studies could provide deeper insights into the impact of PAS on long-term maternal health and neonatal outcomes. Additionally, studies assessing interventions to reduce PAS risk, such as promoting vaginal birth after cesarean (VBAC) in appropriate candidates, could inform clinical practice guidelines.

From a policy perspective, efforts should focus on reducing unnecessary primary and repeat cesarean sections, which remain the primary driver of PAS incidence. National initiatives promoting evidence-based labor management and increasing access to trial of labor after cesarean could help mitigate PAS risk. Additionally, addressing disparities in PAS incidence requires policies aimed at improving prenatal care access for marginalized populations, particularly in high-risk regions. Expanding specialized PAS care centers and ensuring equitable access to high-quality, evidence-based obstetric care, including reducing unnecessary primary cesarean sections, promoting safe VBAC to reduce multiple repeat cesareans, and facilitating timely referral for high-risk pregnancies could help reduce racial and geographic disparities in PAS outcomes.

In conclusion, PAS remains a significant maternal health concern in the United States, with increasing prevalence and associated morbidity. A multidisciplinary approach encompassing clinical, research, and policy interventions is essential to mitigate its impact and improve maternal–fetal outcomes.

## Abbreviations Used

aOR: Adjusted odds ratio
APC: Annual Percent Change
CDC: Center for Disease Control and Prevention
CI: Confidence Interval
COPD: Chronic Obstructive Pulmonary Disease
HCUP: Healthcare Cost and Utilization Project
ICD-10-CM: International Classification of Diseases, Tenth Revision, Clinical Modification
NIS: Nationwide inpatient sample
OR: Odds ratio
PAS: Placenta accreta spectrum
SMM: Severe maternal morbidity
