# Supplementary material for: Update on Trends in Placenta Accreta Syndrome and Its Impact on Maternal–Fetal Morbidity in the United States
Source: Womens Health Rep (New Rochelle). 2025 Sep 24;6(1):988–1000. doi: 10.1177/26884844251378999 (PMC12549190; doi:10.1177/26884844251378999)
Supplement: Supplementary Table S1 [file 26884844251378999_supplementary_tables1.docx]

| **Supplemental Table 1: International classification of diseases, tenth revision, clinical modification (ICD 10-CM) diagnosis and procedure codes used to identify pregnancy-related hospitalizations, placental accreta syndromes comorbidities, covariates, and outcomes** | | |
| --- | --- | --- |
| Variable | Condensed ICD-10 Diagnosis Code | Condensed ICD-10 Procedure Code |
| Pregnancy-related hospitalizations | | |
| Delivery | Z37.x, O80, O82, O75.82  DRG codes: 765–768, 774–775, 783–788, 796–798, 805–807 | 10D00Zx, 10D07Zx, 10E0XZZ |
| Not Delivery (Excluded) | O00–O04, O07–O08 | 10A00ZZ, 10A03ZZ, 10A04ZZ, 10A07Zx, 10A08ZZ |
| Postpartum | Z39.x, O10.x3, O11.5, O12.x5, O13.5, O14.x5, O15.2, O16.5, O24.x3, O2443x, O24.8x, O24.9x, O25.3, O26.x3,  O87.x, O88.x3, O89.0x, O89.1, O89.2, O89.3, O89.4, O89.5, O89.6, O89.8, O89.9, O90.8x, O90.9, O91.x2,  O98.x3, O99.03, O99.13, O99.215, O99.285, O99.315, O99.325, O99.335, O99.345, O99.355, O99.43, O99.53,  O99.63, O99.73, O99.815, O99.825, O99.835, O99.845, O99.89, O9A.13, O9A.23, O9A.33, O9A.43, O9A.53,  O86.29, O86.4, O86.81, O86.89 | 769, 776 |
| Antepartum | O09.x, O09A.x, Z33.x, Z34.xx, Z36, Z32.01, Z33.2, Z33.3,  O10.xxx, O11.x, O12.xxx, O13.x, O14.xxx, O15.x, O16.x,  O20.x, O21.x, O22.xxx, O23.xxx, O24.xxx, O25.x, O26.xxx,  O28.x, O29.xxx, O30.xxx, O31.xxx, O32.xxx, O33.xxx, O34.xxx,  O35.xxx, O36.xxx, O37.x, O38.x, O39.x, O40.xxx, O41.xxx,  O42.xxx, O43.xxx, O44.xxx, O45.xxx, O46.xxx, O47.xxx, O48.x,  O60.xxx, O88.xxx, O90.x, O91.xxx, O98.xxx, O99.xxx, O9A.xxx |  |
| Placenta Accreta Syndrome | O43.2 |  |
| Tobacco use | F17.2xx |  |
| Obesity | E66.x, Z68.30–Z68.45 |  |
| Diabetes | E10.xxx, E11.xxx |  |
| Alcohol use | E24.4, F10, G31.2, G62.1, G72.1, I42.6, K29.2, K70.x, K85.2, K86.0, T51.x, R78.0, Y57.3, X65, Y90, Y91, Z50.2, Z71.4, Z72.1 |  |
| Cannabis use | F12.xx |  |
| Opioid use | F11.xx, F19.xx, T40.xx |  |
| Bipolar disorder | F30.x, F31.x, F34.0 |  |
| Anxiety | F06.4, F40.x, F41.x, F42.x, F43.10, F43.11, F43.12, F43.22, F93.0, F94.0, R46.6 |  |
| Depression | F06.3x, F32.x, F33.x, F34.x, F31.3x, F31.4, F31.5, F31.6x, F38.x, F39, F99, O90.6 |  |
| COPD | I27.81, I27.82, I27.9, J40–J47, J60–J67, J68.4, J70.1, J70.3 |  |
| Asthma | J45 |  |
| OSA | G47.3 |  |
| Diabetes | E10.xxx, E11.xxx, E12.xxx, E13.xxx, E14.xxx |  |
| Hypertension | I10, I11.x, I12.x, I13.x, I15.x, I16.x, I69.x, N26.2, O10.xxx, O11.x |  |
| Heart Failure | I09.81, I09.9, I11.0, I13.0, I13.2, I25.5, I42.x, I43, I50 |  |
| Obesity | E66.x, Z68.3x, Z68.4x |  |
| Gestation hypertension | 'O13' |  |
| Severe Preeclampsia/Eclampsia | 'O11', 'O141', 'O142', 'O15' |  |
| Preeclampsia | 'O14' |  |
| Eclampsia | 'O15' |  |
| Superimposed PE | 'O11' |  |
| Preterm birth | 'O601', 'Z3A1-Z3A36', 'O4201' |  |
| Postpartum hemorrhage | 'O72' |  |
| Intrauterine fetal demise | 'O364' |  |
| **Variables used to create a compose variable – Severe Maternal Morbidity** | | |
| **Acute Myocardial Infarction** | I21.xx, I22.x |  |
| **Aneurysm** | I71.xx, I79.0 |  |
| **Acute Renal Failure** | N17.x, O90.4 |  |
| **Acute Respiratory Distress Syndrome** | J80, J95.1, J95.2, J95.3, J95.82x, J96.0x, J96.2x, J96.9x, R06.03, R09.2 |  |
| **Amniotic Fluid Embolism** | O88.1xx |  |
| **Cardiac Arrest / Ventricular Fibrillation** | I46.x, I49.0x |  |
| **Conversion of Cardiac Rhythm** |  | 5A12012, 5A2204Z |
| **Disseminated Intravascular Coagulation** | D65, D68.8, D68.9, O45.xxx, O46.xxx, O67.0, O72.3 |  |
| **Eclampsia** | O15. X |  |
| **Heart Failure / Arrest During Surgery or Procedure** | I97.12x, I97.13x, I97.71x |  |
| **Puerperal Cerebrovascular Disorders** | A81.2, G45.x, G46.x, G93.49, H34.0x, I60.xx, I61.xx, I62.xx, I63.xx, I65.xx, I66.xx, I67.xx, I68.xx, I97.810, I97.811, I97.820, I97.821, O22.5x, O87.3 |  |
| **Pulmonary Edema / Acute Heart Failure** | I50.x, J81.0 |  |
| **Severe Anesthesia Complications** | O29.11x, O29.12x, O29.19x, O29.21x, O29.29x, O74.0–O74.3, O89.0x, O89.1, O89.2, T88.2XXA, T88.3XXA |  |
| **Sepsis** | A32.7, A40.x, A41.x, I76, O85, O86.04, R65.20, R65.21, T81.12XA, T81.44XA |  |
| **Shock** | O75.1, R57.x, T78.2XXA, T81.10XA, T81.11XA, T81.19XA, T88.6XXA |  |
| **Sickle Cell Disease With Crisis** | D57.x |  |
| **Air and Thrombotic Embolism** | I26.x, O88.01x, O88.21x, O88.31x, O88.81x, T80.0XXA |  |
| **Hysterectomy** |  | 0UT90ZL, 0UT90ZZ, 0UT97ZL, 0UT97ZZ |
| **Temporary Tracheostomy** |  | 0B110F4, 0B113F4, 0B114F4 |
| **Ventilation** |  | 5A1935Z, 5A1945Z, 5A1955Z |
